# Mental Health Services Required after Disasters: Learning from the Lasting Effects of Disasters

**DOI:** 10.1155/2012/970194

**Published:** 2012-07-01

**Authors:** A. C. McFarlane, Richard Williams

**Affiliations:** ^1^Department of Psychiatry, Centre for Traumatic Stress Studies, The University of Adelaide, Adelaide SA 5000, Australia; ^2^Mental Health Strategy, Welsh Institute for Health and Social Care, University of Glamorgan and Ty Bryn, St Cadoc's Hospital, Aneurin Bevan Health Board, NHS Wales, Lodge Road, Caerleon, Gwent NP 18 3XQ, UK

## Abstract

Disasters test civil administrations' and health services' capacity to act in a flexible but well-coordinated manner because each disaster is unique and poses unusual challenges. The health services required differ markedly according to the nature of the disaster and the geographical spread of those affected. Epidemiology has shown that services need to be equipped to deal with major depressive disorder and grief, not just posttraumatic stress disorder, and not only for victims of the disaster itself but also the emergency service workers. The challenge is for specialist advisers to respect and understand the existing health care and support networks of those affected while also recognizing their limitations. In the initial aftermath of these events, a great deal of effort goes into the development of early support systems but the longer term needs of these populations are often underestimated. These services need to be structured, taking into account the pre-existing psychiatric morbidity within the community. Disasters are an opportunity for improving services for patients with posttraumatic psychopathology in general but can later be utilized for improving services for victims of more common traumas in modern society, such as accidents and interpersonal violence.

## 1. Introduction 

Disasters, by their nature, are events that are unanticipated when and where they occur and are situations in which the protective mechanisms of a civil society are particularly likely to fail. Globally, there is an average of at least one disaster every day, the frequency of disasters is rising with climate change, as are the numbers of people involved with increasing population densities but, overall, the proportionate mortality caused by natural disasters has fallen. 

Conversely, the morbidity caused by people's exposure to trauma is rising and a substantial portion of the health effects lies in the psychosocial and mental ill health domains, and this is placing a rising burden on services that are often very stretched already [1]. 

In response, an approach is required that is built of public health interventions for groups of people. Practical and community support are critical in the immediate aftermath of events though rarely should mental health professionals provide that care. In addition a population-based approach enables primary and secondary preventative intervention to be offered and it facilitates evidence-based practice. This paper uses the term psychosocial care to describe these population-based interventions and to differentiate them from the mental healthcare that is provided by primary care and specialist mental health services, usually, later in the trajectory of events. 

There is predictable psychiatric morbidity in populations who are exposed to disasters. The most common effects include distress and grief, and, less frequently, but nonetheless commonly, anxiety disorders and depression [2]. People's attempts at self-medication for the stress, often with alcohol, may lead them into harm and substance use disorders, which may also mask mood and anxiety disorders and postpone their recognition. 

Tol and van Ommeren say [2] “*… mental health and psychosocial support programmes … are increasingly a standard component of humanitarian response.*” Thus, this topic is of global concern. However, the capacities of mental health systems across the world vary widely. While the evidence on the effectiveness of interventions that is available now remains far from complete, it is sufficient to enable us to outline a generic model of care that can be adapted to particular circumstances [3].

This paper outlines how psychosocial and mental health resources should be developed in the setting of particular events that can then be mobilised at times of disaster to deal with an entire population. It provides a framework based on enabling nonmental health trained responders to provide psychosocial care and identifying people who have unmet needs arising from mental disorders on whom to focus the work of mental health services.

This paper reflects on the literature about disasters and the authors' personal experiences following a number of events in which they have been involved. They include the long-term effects of a major naval disaster that occurred in 1964, two major bushfire disasters in Australia, the Iraqi occupation in Kuwait in 1990, the bombings in London in 2005, and the compound disaster of 2011 in Fukushima, Japan. However, the lessons about people's need for psychosocial care and mental healthcare also apply to more common everyday traumas.

Disasters are collective events that evoke a great deal of public concern and distress. The normal methods and structures of civil administration are seriously challenged during and following major incidents [4]. In their wake, major resources are mobilized to assist the survivors. An irony is that smaller scale events occur every day within families and communities. They may be as traumatizing to particular people as are the larger scale events that evoke so much, usually short-term public engagement. But the common small-scale traumatic events, such as, domestic violence and abuse have similar adverse long-term consequences for people's psychological and social wellbeing [5–7]. They attract less concentrated public concern despite there being much to learn from disasters for day-to-day practice and vice versa. 

## 2. The Societal Impacts of Disasters 

The lessons learned from each disaster must inform governments' policies, strategic planning, design and operation of services, and clinical practice so that a cycle of learning and knowledge acquisition continues within all societies and communities. Thereby, people and communities can be assisted to become more resilient and services more appropriate to their needs. Many studies have focused on assessing the prevalence of PTSD among survivors of natural and technological disasters. However, Norris [7] has highlighted in a review of 225 studies that the quality of more recent research is, if anything, declining due to small samples and short followups. This is evidence that knowledge and practice in this field can be repetitive iterations with less than optimal learning from past lessons. Tol and van Ommeren [2] also call for more evidence for psychosocial care, in particular.

Each disaster is unique and poses unusual challenges. Therefore, it is not possible to prescribe in detail simple solutions to the requirements for providing psychosocial and mental health care following them. However, experience, from the 1993 World Trade Centre Bombing, has brought significant improvements in evacuation procedures, leading to the 9/11 evacuations being highly successful [8]. The corollary is that rapid evolution of strategic and operational plans must continue through all phases of each disaster in order to adapt the general plan that was formed beforehand to ensure that it fits the particular circumstances of the disaster with which the responsible authorities are dealing at the time. To this end, planners responsible for the emergency preparedness of many communities, regions, and nations are developing and testing template plans for responding to a range of disastrous situations. Various organizations have provided generic frameworks and/or standards that are capable of adaptation for different societies and which are able to recognise fully people's differing cultures [1, 9, 10].

Often, disasters include single or a small series of incidents that occur suddenly, such as, the bombings in London on 7 July 2005 [11]. Others are characterised by drawn out events, such as, flooding and fires that start suddenly, but may persist for weeks. These types of event cause enormous nondisordered distress, which is an anticipated response that affects a large proportion of the populations affected but also substantial rates of anxiety disorders and depression probably because the traumatic effects persist for many months [2, 12, 13]. Plans for both types of disaster must include plans for responding effectively to the psychosocial and mental health needs of the affected populations. 

One of the major reasons for deaths in disaster is the failure of technology to control the natural environment. In the modern world, we see nature as being a containable and controllable dimension of our existence. Engineering design has done much to manage the forces of nature. People tend to take this for granted so that there is horror when these defenses are breached [14]. The impact of disasters tends to be much more destructive in Third World countries because of the fragility of their engineering infrastructure. 

Often, disasters are major turning points or benchmarks in the history of communities. The Tangshan earthquake that occurred in the height of the Cultural Revolution in the China on 28 July 1976 demonstrates this. 242,000 people were killed and a further 164,000 were badly injured [15]. While the gang of four had a campaign in the media about concern for the victims, they used this as an environment to attack Deng Xiaoping, their political opponent. They were seen by their statements to be callous and indifferent to the suffering of so many bereaved and traumatised people [16]. Within four months, Madam Mao had been arrested and the tyranny of the gang of four had been ended. This was the beginning of the liberation of modern China. 

Disasters are also the turning points in many of the safety measures that are used to protect and organize civilized society. After the sinking of the Titanic, for example, very strict rules were created for the number of lifeboats and buoyancy devices in relation to passengers [17, 18]. The changes in airport security following 11 September 2001 provide another example. This paper advises on certain lessons for mental healthcare that can be derived from past experience and research. 

Disasters are also events in which the survivors are generally less stigmatised than when they are involved in singular events. This is partly because there is an acceptance of the general vulnerability of the community, rather than adverse outcomes being attributed to individual people's lack of resilience [19]. 

One of the greatest challenges is to create awareness among politicians and health agencies about the time course and the patterns of immediate, medium-term and longer term psychosocial and mental health needs of affected populations. In the initial aftermath, there is public outpouring of care and concern. However, communities recover, move on, and forget [20]. But, this is the very time at which the mental health needs of disaster-affected communities pose challenges because a relatively small proportion of survivors are most in need of services that address their longer term adverse outcomes. Their needs interact with the existing morbidity within affected communities, but reshape its presentation and understanding. 

## 3. Types of Disasters 

The type of event is important when considering the rates of morbidity following disasters and designing the types of health services that are required to deal effectively with affected populations. Furthermore, the impact of disasters is changing as our countries become more densely populated. 

Some events are geographically circumscribed. They may have clear margins of demarcation between the people who are affected and those who are not, such as, in major fires. In other disasters, such as, earthquakes and storms, there is a gradient of destruction from the epicenter. The Enschede fireworks factory disaster affected people who came from a defined geographical region [21, 22]. In these circumstances, disaster services should be carefully integrated with the existing primary and secondary health care networks that particular people use at such times. Similarly, the flooding in England in 2007 affected populations in certain parts of the country by invading their homes and workplaces, and the impacts on the affected communities are likely to be long-term. 

Travel accidents and terrorism pose a current challenge. They may affect commuter transport systems, which have localized effects on particular communities, but they may be remote from where the events take place. The Sarin attack in Tokyo and the terrorist bombings in Madrid of 2004 are examples. The significant majority of the people affected are from local communities. Another example is the Clapham rail disaster in London, in which many of the people affected lived in a more distant county that was not otherwise involved in the event [23]. In contrast, if major international transport systems, such as, trains or aircraft are targeted, the survivors come from widely distributed regions and nations. The Bali terrorist bombing is an example. 

Hence, planning for the types of health services required should be cognizant of these substantial differences. After the bombing in Bali, for example, retrieving large numbers of seriously burnt people was an extreme challenge to health systems in Australia. There was no established plan for their retrieval from a place where the health care systems were inadequate. 

Other disasters may affect people from widely distributed geographical regions. The immediate and short-term responses are required at the place at which the event occurs, but short-, medium-, and longer term services are required at a distance or on a distributed basis. In other words, the systems of mental healthcare that are required in the immediate aftermath of the event in which the survivors and the relatives of people who have died congregate are very different to those that are required in the medium and longer terms as people return to their regions of domicile. However, it is important to the continuity of care that the immediate responses and the longer term ones are well coordinated. Thus, unique, but different, outreach systems of care are required in these differing circumstances, and there are challenges to create effective communication systems to sustain individual care plans that may involve many service providers that are separated geographically. 

The impact of a disaster is influenced substantially by the duration of the warning and impact phases. Evacuation systems can be put in place if there is a significant period of anticipation, such as, with storms. 

The terrorist attacks on New York on September 11 were particularly challenging because they affected local communities, tourists, and international business people. However, at least one major company, which was based in one of the twin towers, had a major incident plan that was well planned and rehearsed regularly. While, there were other factors that affected their survival, the staff of that company suffered fewer fatalities; staff knowing what to do was an important factor. 

The major events in Fukushima in Japan in 2011 illustrate another important feature of large-scale disasters, which is that, often, they transcend the traditional division categorization of natural and human-made disasters. The crisis began with an earthquake, which led to a tsunami, the inundation of a nuclear power plant, and radiation hazard. The traumatizing features were multiple, unfolded in full view of the world, and, together, affected directly a very large number of people and an even greater number in Japan and across the world through the economic consequences. The effects on people's mental health are still emerging and are likely to be long term.

## 4. Research into the Psychosocial and Mental Health Impacts of Disasters

### 4.1. Types of Disaster Studies

When considering the prevalence and types of reactions to disasters, it is important to understand the variety of methodologies that have been used. This is because there are so many different approaches that have been used and many have substantial weaknesses [12, 13]. Commonly, there are four sampling methodologies used in disaster research. Each has certain strengths but, importantly, weaknesses too.

#### 4.1.1. Samples of Convenience

Frequently, researchers examine samples of convenience, given the chaos and confusion that occur in the aftermath of disasters. It is difficult to form broad generalizations from these studies. Nonetheless, samples of convenience play an important role in disaster research because, frequently, groups of survivors pose particular challenges with regards to their needs for health services. An example is of the studies conducted after the earthquake in Turkey, which examined various groups, such as, people in emergency housing [24, 25]. 

#### 4.1.2. Treatment Seeking Samples

Clinicians working in disaster settings have often conducted specific examinations of the people who approach them to seek treatment [26]. These studies may assist clinicians to understand when people may present the nature of their complaints and the clinical needs of populations in similar circumstances. 

#### 4.1.3. Longitudinal Studies

Increasingly, longitudinal studies have been conducted, such as, those after the Enschede Fire Works disaster [27]. They demonstrate the patterns of longer term morbidity and are useful in modeling the aetiology of posttraumatic morbidity and how this can be best addressed by health services. They tend to be uncommon in disaster research [28].

#### 4.1.4. Special Subpopulations

Emergency service personnel are an important group who are at particular risk in disasters. A number of studies have identified the impacts of major disasters against the background of the chronic strains of their roles. Children also evoke particular interest. 

A core issue that complicates the interpretation of the data is that, often, the sampling is not representative. Also, there is a tendency to not include the people who are most severely affected because of the demands imposed by involving them in postdisaster studies.

However, some studies, which use stratified epidemiological samples, have looked at representative cohorts with comparison groups [6]. An example is the study of the Yun Nan Earthquake by Cao et al. [29], which examined the populations of three villages at increasing distances from the epicenter of an earthquake in China. The people in these villages were compared with those in a village unaffected by the earthquake that was demographically very similar. This approach addresses the issue of exposure as well as the background problem of the prevalence of morbidity in the community by presuming this to be the same for people who live in the unexposed village. 

### 4.2. The Psychosocial and Mental Health Impacts of Disasters

Disasters and terrorism capture human attention and concern, but public interest tends to be relatively short lived [20, 30]. Often, their long-term morbidity is under-estimated by planners and health and social welfare providers [31]. While concern for the survivors and the relatives of people who are killed, seriously injured or missing, is potent in the immediate aftermath of these events, it tends to be forgotten in the longer term. The impacts on the lives and mental health of the staff who care for people who suffer profound injuries in disasters and long-lasting impairments afterwards are also neglected. A meta-analysis documents population-level behavioral and psychological consequences of terrorist incidents [32].

The Inter-Agency Standing Committee (IASC) is formed by the heads of a broad range of UN and non-UN humanitarian organisations. In 2007, it published Guidelines on Mental Health and Psychosocial Support in Emergency Settings [9]. That document segments the psychosocial and psychiatric consequences into groups. Tol and van Ommeren [2] highlighted that there are a range of consequences of disasters. They remind us that “ *… mental health practitioners in humanitarian settings frequently encounter people with severe pre-existing neuropsychiatric disorders …*”* and *“* … disasters and armed conflicts … impact the social conditions that shape mental health, through increased poverty, threats to human rights, domestic and community violence and changes social relations.*” In summary, these groups include people who have:

1. pre-existing social problems (e.g., extreme poverty, belonging to a group that is discriminated against, marginalized, or politically oppressed);
2. emergency-induced social problems (e.g., family separation; disruption of social networks; destruction of community structures, resources and trust; increased gender-based violence);
3. emergency-induced distress (e.g., grief, anticipated or nonpathological distress and distress in which the symptoms may appear similar to those of acute distress disorder, posttraumatic stress disorder, and/or depression);
4. emergency-induced mental disorders: depression and anxiety disorders, (including acute stress disorder and PTSD), substance misuse, and sustained personality changes;
5. humanitarian aid-induced social problems (e.g., undermining of community structures or traditional support mechanisms);
6. humanitarian aid-induced psychological problems (e.g., anxiety and depression due to lack of information about food distribution or due to poor management of the information that is provided);
7. pre-existing mental health or psychiatric problems (e.g., severe mental disorder, alcohol abuse).

It is important to have an overview of the wider panoply of risks and effects that emergencies, major incidents, and disasters of all kinds may have because, very importantly for policy, service planning, and clinical practice, many people fall into more than a single group. Furthermore, the groups are by no means separate because the effects of one type of consequence may act as risk or exacerbating factors for others. Recent experience highlights the overlaps. Extensive flooding in parts of the UK in 2007 and Australia in 2011 rendered the homes of a large number of people uninhabitable. This posed immediate problems for their safety and domicile and many risks to the health and mental health of the public [12, 13, 33]. It also threatened essential local services (e.g., power supplies, sewerage, and availability of potable water) because they were engulfed or because staff of those services were affected. 

Thus, many people who were not directly involved in the emergency also had the services on which they depend affected or put at risk. The risks to essential services include the preoccupation of staff with other matters, problems with transport and locating people. All of these effects become secondary stressor, which, when persistent, as in the case of flooding, are potent perpetuators of people's distress and provoke delayed mental disorders such as depression [12, 13, 33]. They pose threats to care, including mental healthcare, in affected communities. People who are not directly involved in disasters are also at risk, if they require continuity of mental health services. 

A specific challenge in disasters is to assess the health and welfare needs of first responders and staff of the emergency services involved in rescuing survivors and retrieving the bodies or remains of the people who have been killed [34]. Emergency service personnel are trained to carry out this work and, but many fewer have effective support networks to assist them to deal with the adversity. Staff training should include structured systems to build and support the resilience of staff. Staff who care for people who have multiple or long-term injuries also require consideration. Often, the capacity for support structures to contain and deal with these circumstances is overwhelmed by events. 

The effects of emergencies on the needs of people for psychosocial and mental health services require planners to consider the practical requirements of four main groups of people as indicated in Table 1.

All of these groups (see Table 1) acquire psychosocial needs and a smaller but substantial proportion may develop mental disorders. Planners must take account of each one. They must also be aware of the ripple effects whereby the pool of people involved tends to grow by association and of the needs to coordinate planning of immediate and medium and long-term responses sometimes over wide geographical areas depending on the nature of the events and the origins of the people involved. These challenges require appointment of knowledgeable and experienced specialist advisers who respect and understand the existing healthcare and support networks for the people who are exposed to disasters, as well as recognising the cultural preferences of people who are affected. 

Positively, disasters can be used as circumstances for creating much greater awareness and skill about psychopathology in communities and as examples of the importance for all mental healthcare services providing planned and coordinated programmes of care. Training mental health professionals to provide responsive services at the time of a disaster can be utilized later to deal with the more ubiquitous and day-to-day trauma that exists with great prevalence in all countries. Presently, much of this morbidity is unrecognized and poorly treated. 

## 5. Lessons from Research 

### 5.1. The Reactions of Survivors and Professionals

In the immediate aftermath of disasters, survivors tend to seek out their own networks of support, if they are available. This desire should be recognised and actively supported. The majority of people has a natural stoicism and tends to minimize their focus on the psychosocial impact and anticipate improvement in their distress. On the other hand, professionals usually feel an obligation to assist and often encourage people to seek counseling. However, they should focus more on individual person's distress and must guard against exaggerating the psychosocial and mental health risks or apprehension about survivors ability to cope. This conflict means that it is critical for survivors' perspectives to be respected and addressed. 

On the other hand, tendencies to exaggerate personal hardiness and the stigma and shame of seeking help with social, psychological, and mental health needs must be carefully managed in any service intervention provided. People who are involved (staff as well as survivors) may use avoidance as a psychological strategy for dealing with their distress. 

Critical consequences that must be planned for when delivering health services include the confusion that people may experience about the meaning of their experiences and the frequency with which people who are psychologically distressed present with physical symptoms. This occurs particularly often in war veterans but also after disasters. McFarlane and Papay et al. [35] studied the pattern of presentation of firefighters to their general practitioners and their histories of health complaints after the Ash Wednesday bushfires. Many of these men had attended their general practitioners to complain of a range of physical symptoms, which they experienced to a significantly greater degree than another group of fire officers who had similar rates of injury and exposure but did not have posttraumatic stress disorder [36]. Often, the general practitioners did not recognise the symptoms as being indicators of a possible psychiatric disorder. 

The importance of this section is that it alerts planners to the importance of designing services, which take into account the integrated physical and psychological needs and preferences of affected people. It also reminds us that any services offered should be based on planners and practitioners sound understanding of the issues that arise from different cultural and societal preferences, expectations, and needs.

### 5.2. Resilience

While there is longstanding evidence that a substantial number of people who are exposed to disasters develop psychopathology for short, medium, or longer periods of time, there is growing international interest in how and why many and so more people who are involved in disasters do not develop mental disorders. 

There is an increasing evidence that many people appear to be resilient to the deleterious sustained psychosocial impacts that are experienced by a most of the people who are involved [37]. In addition, there is also a small but slowly increasing number of studies showing that some people may suffer distress in the short-term but also derive certain psychosocial developmental advantages too. One of the intentions of this work is to learn lessons for how we might reduce the risk of mental disorder that is imposed by people's exposure to a wide variety of stressors, and it is leading to the development of public mental health services.

Sapienza and Masten [38] and Masten and Narayan [39] provide summaries of the development of the concept of resilience and research evidence in two recent papers that focus on children. Other recent work has researched the resilience of groups of people and communities. This is termed collective resilience as compared with personal resilience, which covers the resilience of particular people. It showed that strangers who are thrown together by circumstance to face disaster as a group often develop collective resilient patterns of behaviour very quickly in their immediate response to unfolding major incidents and that this phenomenon is not limited to communities or groups of people who are well-known to each other. These patterns of behaviour were found in the people caught in the underground rail carriages in the bombings in London on 7 July, 2005 [40]. 

Personal and collective resilience offers psychosocial advantages that buffer the ill effects of disasters on people's mental health. This prompts a question about whether it is possible to prepare people and communities for the possibility of disaster in ways that strengthen them and mitigate the risks of mental disorder without provoking widespread fear and anxiety. As yet, there is little research into the effectiveness of resilience building [33]. However, presently, programmes are in hand in a number of countries that are intended to promote the resilience of people and communities. 

While promoting resilience is a broad societal matter and not a direct call upon health services, there is an increasingly popular argument for including it in the spectrum of psychosocial services that are required in mature cross-agency plans for responding to disasters [33]. 

### 5.3. Psychopathology

The most consistent finding from disaster studies is that intensity of people's exposure is the critical determinant of their risk of psychopathology [29, 41]. Exposure is defined by a combination of the threat to particular persons and groups of people and the losses that they sustain [42]. Measuring exposure is not a straightforward issue. Sometimes, it is defined by a group in terms of being a member of the community that has suffered greatly or according to personal loss. 

A difficulty in interpreting findings from research is that exposure is affected by other factors that modify, intensify, or buffer its affects. Recently, for example, meta-research has shown that availability of psychosocial support or otherwise has a very substantial moderating effect on the risk generated by exposure [43]. This finding emphasises the importance of planning services in advance. However, it is difficult to directly compare many studies because of the different time intervals that have elapsed from the point of the disaster to the point of the investigation [12, 13]. In all populations exposed to major incidents, there is an expected process of natural remission and this impacts on the prevalence rates according to the time that has elapsed after an event. 

Disaster is only one type of traumatic experience that can affect people's lives. One way of understanding the comparative effect of different categories of traumatic events is to look at general community stratified population samples and examine the prevalence of disaster exposure. The National Mental Health and Wellbeing Survey conducted in 2007 [44] in Australia looked at 10,600 people. PTSD (diagnosed using ICD-10 criteria) was found to be the most common anxiety disorder. One of the problems with the methodology in these studies is that they ask people about only one event. Among the Australian population, 20% of men and 13% of women had been exposed to disasters. Compared with the rates of PTSD following most of the other major stressors that were examined in this population, the rates were significantly less than those after events involving interpersonal violence. The 12-month prevalence among the disaster-exposed population using DSM-IV criteria was 2.3%. Although this is a small percentage, it reflects a long tail of effect because for many of the people the disaster may have occurred many years previously. 

As set out above, there are a substantial percentage of people in any disaster-affected community who are already suffering from psychiatric disorders as well as those who become unwell due to the disaster. This frames an enduring challenge to disentangle the interaction between these two groups. In other words, the percentages of people who have never previously suffered from psychiatric disorder and who emerge with a diagnosis after trauma are of particular interest. The way in which the existing psychopathology is changed in terms of its phenomenology and longitudinal course is an equally critical question. Most disaster studies to date have not made this differentiation. This latter question is of particular importance in respect of the observations of Kessler et al. [45] in the National Comorbidity Study. It demonstrated that PTSD could be a disorder that runs a significantly chronic course in approximately 40% of people who are so diagnosed. The positive perspective is that, in 60%, the disorder ameliorates by 72 months and community-based treatments appear to have beneficial effects. Long-term studies of disasters are few with the 33-year followup of the Aberfan mudslide disaster showing that 46% of the childhood survivors had had PTSD at some stage, and 29% had that diagnosis at followup [46]. The Piper Alpha oilrig disaster of 1988 ten years later found that, 21% of the survivors was found to have PTSD [47]. Studies, such as, these indicate that people who have had a high intensity exposure have a significant risk of prolonged and enduring morbidity. This is the challenge for health services namely, anticipating, identifying, and treating people in the long-term as well as the short-term need following these events. 

While studies measure anxiety disorders, such as, PTSD, fewer measure the prevalence of mood disorders though the studies that do show that depression is commonly comorbid with PTSD or occurs in the absence of PTSD [12, 13, 48, 49]. While particular interest is focused on PTSD following disastrous events, there is, now, an increasingly substantial body of evidence demonstrating that the whole range of psychiatric disorders emerge with an increased risk following these events. Bonanno et al. [50] offer an authoritative review of the literature relating to the psychosocial and mental health costs of disasters. They say, “*People exposed to disaster show myriad psychological problems, including PTSD, grief, depression, anxiety, stress-related health costs, substance abuse, and suicidal ideation.*”

A study that one of us [51] conducted looking at 200 motor vehicle survivors, a substantial group developed major depression alone in the absence of PTSD [52]. Equally, many people with PTSD had significant degrees of comorbidity. Similarly, the growing volume of good quality research into mental health of the UK armed forces is beginning to reveal that the most common disorders among veterans who have served in Iraq are depression (53% of those with a mental disorder), anxiety disorders (18%) PTSD (16%), and substance misuse (12%) [53]. These findings emphasise the importance of research in future examining the broad pattern of morbidity [12, 13, 48].

## 6. Effective Interventions 

### 6.1. An Evidence-Informed Model of Care

The interventions recommended by IASC and the Sphere Project's standards are intended for adoption in all parts of the world, societies, and cultures [9, 10]. They are based on the best available evidence. It is imperative that frameworks for disaster mental healthcare are adaptable. A recent paper states that “*Mental health care is desperately needed throughout the developing world*” and it reports that Aceh province in Indonesia is testing a novel approach, which minimizes reliance on psychiatrists [54]. The guidance from IASC, Sphere, and NATO is intended to be applicable in a wide range of differently resourced countries.

Tol and van Ommeren [2] draw attention to the four layered pyramid of intervention that these sources commend comprising of psychosocial care for most survivors and their relatives delivered by nonmental healthcare practitioners combined with mental healthcare that is targeted on people who have diagnosed disorders that is delivered by people who are trained to do so [55]. They draw attention to a recent review of the grey literature that has compared currently popular practices with the evidence for effective practices. They point out that the most rigorous evidence supports specialised practices for intervening with people who have PTSD and depression.

Other bodies have recommended similar approaches to intervention that are intended to be economical with precious trained mental health practitioners who are often of limited availability in many countries. One such approach is the strategic stepped model of care that has been adopted as nonbinding guidance by NATO [1]. It is also based on the best available evidence. Forbes et al. [55] have advocated a stratified approach to psychosocial and mental health care. In 2009, Williams and Bisson brought together work undertaken in Europe with the NATO Guidance to produce a set of principles that is informed by the best evidence available.  Table 2 illustrates a stratified model of care [56].

### 6.2. Intervening in the Rescue and Immediate Recovery Phase

There is a growing professional consensus and slowing rising evidence that a series of psychosocial interventions should be applied immediately after disasters. This approach is based on the principles of psychological first aid. Recently, the WHO published its guide on PTSD for field workers [57].

The philosophy for the psychosocial and mental health interventions is the PIES model that has been developed from the military, namely that services provided should be based on their proximity, immediacy of response, expectancy of recovery, and simplicity. 

It is critical is to make people feel safe and to assist in decreasing their anxiety immediately after a disaster impacts. It involves providing information and dealing with the immediate survival, welfare, and humanitarian needs, if requested. People should be provided with reassurance and physical comfort. Those in pain from injuries should be given good pain relief. 

However, providing immediate counseling risks creating a false belief that there will be no long-term morbidity and, furthermore, counseling immediately after disasters has not been shown to be effective. So, it is important to manage carefully access of counselors to survivors. This involves vetting offers for help and credentialing service providers. It is important that there is active interaction between disaster services on-scene and the staff who provide inpatient care for injured persons at a distance because, otherwise, people who require psychosocial support may slip through the usual channels. 

In this phase, some people may develop an acute stress reaction, which may be termed acute stress disorder (ASD) if the experiences are severe and cause dysfunction. On first examination, people who have this disorder may experience symptoms that are similar to those of distress, on one hand, and, on the other hand, those experienced by people who suffer PTSD later. Therefore, great care is required in distinguishing people who are temporarily distressed from those who have ASD and, after a month, PTSD. The most reliable feature is how well people recover over time. Distressed but resilient people recover quickly but may experience quite debilitating experiences for a while. People with an ASD recover more slowly. Progressive mitigation or diminution of their intrusive recollections of the disaster differentiates people who develop PTSD from those who do not. The definition of PTSD precludes its diagnosis until a month after the event. These findings have led the National Institute for Health and Clinical Excellence in the UK (NICE) [58] to recommend that a stance of watchful waiting be adopted for around four weeks after the start of events before treatment for PTSD is initiated. During the interval, survivors should be offered support that is based on the principles of psychological first aid [59] and further assessment. Therefore, one of the primary aims in this phase is to screen the population and make some assessment of people who are at particular risk in the future. However, earlier treatment should be provided if people's distress is unusual or particularly intense. 

In the rescue and early recovery phases, it is best to use local health providers, if possible, because they are embedded in the affected communities, trusted, and know the culture and local circumstances. During this phase, it is also critical to develop a training programme for general practitioners who are likely to be the contact points for people who require long-term services. Even if they are not particularly skilled in the mental health domain at this point, they are the people who tend to be consulted by survivors in the aftermath of the disaster. They are well placed to distinguish people who require psychosocial care from those who need specialist mental healthcare. 

## 7. Developing Services 

### 7.1. Policy Development

The services, including the mental health services, that are required following a disaster only work effectively if planners anticipate the need for them, understand the dynamic shifts in need that occur with the passage of time, and are clear about how these services are to work with other agencies that offer psychosocial responses. 

Mental health services for moderate- and large- scale emergencies should be well integrated with humanitarian aid, welfare, and psychosocial care in disaster response plans. This requires that lessons learned through research and experience are translated in integrated ways into policy at four levels:

1. government policies;
2. strategic policies for service design;
3. service delivery policies; and
4. policies for good clinical practice.

Each of these four levels of policy should be influenced by the kinds of evidence that this paper summarises. Policy at each level should be ethical. There are important roles for practitioners who are skilled in mental healthcare and experienced and trained in disaster management to provide advice to the authorities as they develop each of these aspects of policy and conduct operations in the face of disaster. The principles developed by Williams et al. [56] cover all four policy levels. Cultural and ethical values should permeate each level of policy and planning. 

Government policies are required to set the aims and objectives for psychosocial and mental healthcare responses. They should specify the requirement for services to be designed, developed, and delivered that offer mental healthcare that is integrated into all disaster response plans. The responsible authorities should bring together evidence from research with expert consensus, their knowledge of the country, its society and cultures, and the profile of risks to design services. They should plan programmes for managing the performance of the services in meeting the objectives that are identified for them. 

Service delivery policies concern how particular services function and relate to their partner services and how affected populations are guided into and through them according to the evidence and awareness of the preferences and needs of people who are likely to use them. Therefore, service delivery policies include evidence- and values-based models of care, care pathways, and protocols and guidelines for care as well as processes for demand management, audit, and review. Policies for good clinical practice concern how clinical staff take account of the needs and personal and cultural preferences of patients, deploy their clinical skills, and work with patients to decide how guidelines, care pathways, and protocols are to be interpreted in individual cases. 

Major incident mental health services should be built upon the existing clinical skills and preparedness within each community to deal with day-to-day disasters. Furthermore, the services that provide psychosocial support and those that provide mental healthcare should be an integral part of the major incident or disaster plans for every community. This challenges community leaders' planning and training and raises their needs for knowledge and skill. One way in which the latter task can be approached is by ensuring that the skills that are developed are made available within communities by, for example, the mental health services being available to the survivors of, for example, motor vehicle accidents, house fires, criminal assaults. 

Additionally, there are phases and time windows that are critical to service planning, design and delivery as Williams et al. [56] concur. 

Planning in advance of warning of possible disaster should be ubiquitous. It should cover five domains of service requirement:

- medical rescue and immediate intervention services;
- continuing physical healthcare;
- recovery and rehabilitation services;
- business continuity for preexisting services for the general population (which may, otherwise, be depleted as a consequence of disasters with secondary consequential affects on communities);
- psychosocial and mental health services.

Demands vary significantly across the different timescales and narratives of each disaster. Thought is required about the profile of services required as a disaster unfolds and in the rescue, recovery, and restoration phases. The need for medical services is greatest in the early phases. By contrast, the specialist mental health services are most required later. Psychosocial services are required in the immediate aftermath and recovery phases and have a continuing contribution into the restoration phase. 

### 7.2. Time Windows for Service Planning

#### 7.2.1. The Predisaster Phase

In the predisaster phase, it is critical to ensure that each community has a plan for providing psychosocial and mental healthcare after disasters and that the plan includes immediate and medium- and long-term components. This means that these plans should be flexible and responsive to differing types of disaster by, for example, including the means to alert distant communities to the requirement to plan medium- and long-term responses if the people involved come from wider rather than local geographical areas. The problem is that disaster plans are too often designed around the most recently impacting event, which, in probability, may not be the event that occurs next. It is a challenge to maintain a pool of disaster experience and of staff who are interested in the absence of a real threat. 

It is a challenge to involve mental health services at this stage. Planners tend to see the critical issues as being emergency surgical services and hospital management plans. While those facilities are critical, mental health services should be given an enhanced priority because failure to include them in this stage of the planning means that it is harder to achieve their engagement later. This has implications for training. Decisions of this nature tend only to be made if the government policies include them, and mental healthcare is explicitly specified in strategic designs for services. Otherwise, the resources required, as predicted by appropriate appraisals of the risks, may not be forthcoming. 

Practice in all phases must be ethical, and this calls for a framework of public health ethics (as compared with personal health care ethics) to be adopted during the predisaster planning phase. An example of such a successful framework is provided by the work on public health and policy ethics conducted by the UK government in its preparations for an influenza pandemic, which was tested in 2009-10. Additionally, the Madrid Framework can be used to test how well policies deal with the values and evidence that are inherent in designing and delivering services [56, 60].

#### 7.2.2. The Disaster or Incident Warning Phase

Plans should be put into effect during the period when there is an increased risk of disaster, such as, the summer in bushfire prone zones or during the period when a weather system is emerging as a major threat. This requires acceptance of the heightened danger at all four levels of policymaking and practice. Otherwise, denial results in loss of time that is vital for detailed planning. This occurred in the cyclone that devastated Darwin on Christmas Day in 1975. The disaster warnings were largely ignored because, on 24 December, everybody was busy with their Christmas preparations [61]. Similar denial took place before the floods in the Netherlands of 1953. 

Taking this advice means accepting that there are many instances in which a warning arises, but no disaster follows. it is critically important to be adaptive and for risk appraisal to be flexible during this period because each event poses new challenges. 

#### 7.2.3. The Phase of Disaster Occurrence

As disasters unfold, there is often little that can be done. There are many events, such as, the Kobe earthquake [62], which occurs in such a brief time frame that no action is possible. Other disasters, such as, fires and floods, have a more enduring course. It is critical to be tolerant of loss of control during this stage, and face the sense of helplessness and unpredictability of chance. 

Often, the immediate impressions of a disaster scene for members of the public and staff of the rescue services who are first to arrive are one of such utter chaos and devastation that it is difficult to know where or how to begin work. That experience is immobilizing, and anticipation of what the scene may be like should be built into training along with sound leadership if rescue teams are to acclimatize and begin work rapidly. The terror impact of the attack on the World Trade Centre Towers, for example, was dramatic and the survivors struggled to express their feelings in a way that was understood [63]. 

At these early times, it is not unreasonable for people who are in emergency service roles, such as, ambulance services staff and firefighters, to behave in self-protective ways, rather than taking needless risks. High levels of risk were run by fire officers who went into the World Trade Centre on September 11 because they failed to adequately assess the potential for the buildings to collapse. Survival demands flexibility of risk appraisal and the ability to switch into rescue mode when this becomes a viable proposition. 

Paradoxically, one of the difficulties for staff who come to disasters later in the aftermath is that the awfulness and horror have abated. Often, there is a dreadful silence and stillness in the aftermath of destruction. The contrast between the aftermath and the event often makes it very difficult for those people who have not experienced a disaster to understand the horror and the awfulness of what has been experienced by survivors and the staff of the rescue services. 

One of the predicaments of disaster survivors is that they have been through a time warp of horrors which people not present struggle to grasp. Thus, it is easy for the psychosocial and mental health issues to be ignored in this chaos. 

#### 7.2.4. The Rescue Phase

In the rescue phase, it is critical to assess the assets and resources that are available within the community to address the needs of the population. While the focus is on attending to the acute physical needs of affected people, it is also important to make a preliminary assessment of the survivors' potential long-term needs [64]. Often, new leadership structures emerge in communities as they deal with the unpredictability of the emergency. Acceptance of the fluidity of this situation is a critical part of the capacity of the responding services to face the threat and cope in the disorganisation that follows traumatic events. Sometimes, there is a conflict for staff of the health services between attending to the needs of their families and going to work as a rescuer or health provider. This dilemma is most acute for the emergency service workers, and they require continuing contact with their families, good leadership, support, regular rest, food and breaks, and engagement as members of teams if they are to give of their best. 

Knowing about how people behave in the face of disaster and the longer-term potential for psychosocial effects is important not solely within the health services at this time, but when providing support for and understanding the behaviour of survivors. This frames another important item for training staff, including, particularly, major incident commanders (MICs), well before events occur. 

As events unfold, identification of survivors and their needs for acute physical care is an immediate priority. Threat to life is the immediate priority and threat to limb and other morbidity follows. While the prospects for people having immediate needs for psychiatric care are small (estimated as 1 in 1,000 survivors), it is important to establish if any survivor is in immediate need of specialist mental health services. Similarly, few people's behaviour is grossly disorganized. Often, managing staff and volunteers who want to help is a more substantial challenge. 

Well before events occur, planners should respond to their obligations to rescuers and relief workers by providing them with effective leadership, training, supervision, defined expectations, and support and these functions must be available to staff throughout their work in an emergency. It is important to assess if the size and impact of the disaster require reciprocal assistance from similar services from outside the immediate area to augment local capacity. Plans for reciprocal responses in major emergencies must be in existence prior to any disaster. 

#### 7.2.5. The Medium-Term Recovery Phase

The central role of primary healthcare services should be considered carefully in the recovery phase. Good quality care for physical injuries and adequate pain relief can do much to benefit people's psychosocial states. All psychosocial and mental health services should be integrated with the primary healthcare services and be placed in similar locations so they do not compete. Continuing professional support and education should be provided. 

The mental health practitioners who deal with traumatized survivors and their relatives in the medium-term should be well trained in evidence-based assessment and treatment skills that are required for dealing with posttraumatic mental disorders of all types including depression and anxiety disorders. They should use a multidisciplinary approach and have strong links with the primary healthcare networks that recovering people continue to require. The staff who provide these services should be aware of the interaction between the emerging posttraumatic morbidity and existing psychiatric morbidity within the community. Consultants should be involved in monitoring the emerging morbidity and ensuring that groups of people who are at risk receive services. Planning for future service development should be continuous. 

Experience shows that there is poor uptake of specialist services in the mediumterm after disasters. This requires plans for active outreach that is based on the screening that has been conducted in the previous phase to define the populations that are at risk. Addressing communities' mental health literacy is another critical challenge at this point because of the divergence of the beliefs. The population should be monitored for behaviors, such as, self-harm and patterns of substance use, that may indicate risk indirectly and depression and anxiety disorders. Also, this is the time when other events in civilian life may raise the risks of re-traumatisation for fire, ambulance, and police officers, for example. 

#### 7.2.6. The Long-Term Reestablishment Phase

Throughout all phases of recovery, practitioners should ensure that people who have been involved in disasters have optimal outcomes [65]. Although the number of people who develop persistent mental disorders is relatively small, their needs may be substantial and last for lengthy periods. Some people require maintenance treatment. However, there is a common tendency to withdraw services prematurely. Additionally, there may be an interval, sometimes an extended interval, before some people develop disorders, such as, depression and PTSD [66]. Thus, new cases tend to emerge for many years after disasters. This means that specialist services are likely to be required to address the needs of the survivors of the many lesser disastrous events as well as the major disasters that have befallen the population in the same geographical area. 

After any disaster, there is always the requirement to identify what has worked and what has gone wrong. Reviews should be initiated during the earlier phases but become the focus of particular consideration during the long-term phase. There are lessons to be learned for policy, planning, organisations, leadership, and management of responses. The reestablishment phase provides opportunities to review and address the clinical skills of the professional staff of the mental health services with a view to ensuring that they are adequately trained, and that emerging professional lessons have been identified and learned. 

#### 7.2.7. The Chronic Posttraumatic Reestablishment Phase

General public interest in disaster-affected communities dies quickly. On the long term, the mental health services can play a critical role in maintaining recognition of the continuing additional needs of affected communities. 

The staff of the mental health services who are involved in responding to disasters should also actively manage reintegration of the specialized services with the mainstream structures [67]. Often, additional funds that were made available to discharge disaster response services are withdrawn. This presents challenges because the people who have long-term mental health needs may still require active interventions [68, 69]. All-too-often, the prevalence and impacts of past and future disasters and other major incidents within affected communities are underestimated as is the role of the psychological trauma that stems from disasters in determining the patterns of background psychiatric morbidity in affected communities. 

In some disasters, there is also the important challenge of dealing with coroners' courts, legal systems, and compensation claims. The cumbersome delays and the procedural slowness of legal processes can be a substantial cause of further psychosocial aggravation for survivors. 

## 8. Conclusion 

Looking back, there has been a striking, rapid, and dramatic change in scientific understanding of and public approaches to recognising and managing the psychosocial trauma that may occur as a consequence of disasters. The position taken in this paper is that psychosocial and mental healthcare services should be planned and actively integrated components of all disaster relief and broader healthcare responses.

This requires attention to learning the lessons from research and from survivors' stories about the requirements for psychosocial care of populations of people and mental health services for people who develop depression, anxiety, and/or substance use disorders. There is a strong consensus in the evidence about the types of disorders that people may suffer after disasters. The evidence of effectiveness is stronger for intervening with people who have disorders, though the rising interest in public mental health should propel more research into the needs for and effectiveness of psychosocial care. 

The WHO has espoused the principals for immediate psychosocial care, and there is a strong expert consensus on basing this work on psychological first aid. 

Critically, achieving what is required also requires continuing negotiation with policymakers, managers and clinical staff of all relevant disciplines and backgrounds. Often, emergency preparedness managers and emergency service leaders welcome greater knowledge of the psychosocial and mental health impacts of disasters and that evidence-informed expert consensus frameworks of response that have great similarity are now available. 

The challenge of maintaining the skills developed in the aftermath of the disaster is only met if service delivery models and clinical skills are extended into other settings. The WHO Burden of Disease has emphasised the importance of motor vehicle accidents as a critical cause of morbidity. Furthermore, people who are chronically mentally ill are often the victims of trauma and violence. Thus, this paper could be considered as describing a microcosm of mental healthcare that should allow lessons to be learned for developing services for people who are exposed to less extreme events. The skills learnt by staff of the mental health services can then be effectively transmitted into their work with the survivors of small but no less painful day-to-day traumatic events. Reciprocally, that advance will provide critical skills among managers and practitioners to meet the needs of their community if it were to be affected by a disaster. 

Governments should lead by actively engaging in continuing policy development to take account of our emerging knowledge. At the same time, people who are involved at each of the four levels of policy require active support and assistance. Practical steps that can assist in these regards include creating centres of excellence, such as, the WHO centre in Kobe. Also, developing collaborative associations with researchers and colleagues across the world is vital as is developing education in a variety of institutions and professions that build upon our accumulating knowledge. Only then will systems have been created that are based on cycles of improvement in which the lessons from disasters assist in better management of the survivors of the large, small, and personal disasters that occur daily in all societies. 

## Figures and Tables

**Table 1:** Groups with different mental health needs following disasters.

(i) People who are at risk of distress, mental health problems, and	
mental disorders, principally anxiety, depressive, and substance	
use disorders, consequent on their direct and indirect	
involvement in events and who present new and additional	
demands on mental health services.	
(ii) People who have continuing needs for mental health services	
for preexisting conditions, but whose care is threatened	
by challenges to the “business continuity” of preexisting	
mental health services consequent on network and community	
dislocation.	
(iii) People whose involvement in an emergency provokes or	
precipitates the relapse of a preexisting mental disorder.	
(iv) People who are responders and whose mental health might be	
put at raised risk consequent on their work.	

**Table 2:** Interventions in the aftermath of disasters.

Level	Intervention	Target population	Examples of interventions	Interventions conducted by
1	Psychological first aid (PFA)	Most of the people who are affected	Restoring immediate safety Restoring contact with loved ones	All responders and aid workers
2	Community development	Communities after large-scale events	Schools, sports, meetings, newsletters to unite groups of people	All responders and aid workers
3	Skills for psychosocial recovery (SPR)	People whose distress is sustained by bereavement or secondary stressors	Brief needs assessment	Healthcare practitioners and workers trained in the skills
			Problem-solving	
			Social support	
4	Psychosocial interventions for medium- and long-term problems	People whose distress is sustained and associated with functional impairment	Trauma-focused cognitive behaviour therapy	Staff of mental healthcare facilities
